# Neglected Tropical Disease Control and Elimination: Is Human Displacement an Achilles Heel?

**DOI:** 10.1371/journal.pntd.0003535

**Published:** 2015-03-19

**Authors:** Kaylee Myhre Errecaborde, William Stauffer, Martin Cetron

**Affiliations:** 1 Veterinary Population Medicine, University of Minnesota, St. Paul, Minnesota, United States of America; 2 Department of Medicine Division of Infectious Disease, University of Minnesota, Minneapolis, Minnesota, United States of America; 3 Global Migration and Quarantine, US Department of Health and Human Services, Centers for Disease Control and Prevention, Atlanta, Georgia, United States of America; Swiss Tropical and Public Health Institute, SWITZERLAND

## Introduction

The United Nations High Commission for Refugees (UNHCR) has estimated that over 40 million people [1] are currently displaced and have variable access to health care in the country in which they reside. Populations displaced by conflict are largely disenfranchised, and high prevalence of neglected tropical diseases (NTDs) has been documented [2]. NTDs generally affect the least advantaged people in poor societies—populations with little voice or representation. These already susceptible people become even more vulnerable when forced from their communities as internally displaced persons (IDPs), refugees, or forced migrants. To further complicate matters, many of these people of concern are under 18 years old. Children experience the greatest risk and suffer the most consequences of NTDs. As marginalized populations flee from conflict or environmental catastrophe, they are often burdened with insidious NTDs ranging from asymptomatic to overt and debilitating disease. Many suffer from chronic consequences such as malnutrition, growth stunting and developmental delays, inhibiting chances for sustainable livelihoods and making it less likely that they will successfully overcome the adversity of displacement.

The World Health Organization (WHO) has defined 17 key neglected diseases, but several others exist [3]. These diseases are highlighted in Millennium Development Goal (MDG) 6, which aims to combat HIV/AIDS and “other diseases,” of which the NTDs are discussed at length [4,5]. It is the intent of these authors to raise the awareness of readers, and argue that inclusion of these displaced populations in preventive chemotherapy (PCT) programs and multi-model community-based interventions is not only necessary for sustained success of NTD control but is also a moral imperative.

## Background

Evidence for the association between poverty and NTDs is well documented [3], as is the evidence that human civil conflict, such as war, interrupts infectious disease control and creates opportunities for transmission and outbreaks [2]. However, the impact of human displacement on the epidemiology of NTDs amenable to PCT is lacking. However, it is clear even from the sparse literature that NTD epidemiology is affected by human displacement. Saker et al. mention the appearance of the first human African trypanosomiasis (HAT) cases in southern Ghana owing to population movement [6]. In addition, Louis et al. describe HAT being brought to the suburbs of Kinshasa, Democratic Republic of Congo (DRC), by people fleeing war in HAT endemic provinces [7]. These are just a couple of concrete examples and this pattern is undoubtedly replicated with many NTDs, particularly those that, like most helminthic infections, do not need specific vectors [8]. Aagaard et. al eloquently argue that human migration is a major factor in the introduction, and re-introduction, of NTDs [2]. The risk for NTD expansion is greatest when displaced populations are forced to set up temporary living situations where sanitation is inadequate. Displaced populations frequently reside in warm and humid environments, and the combination of poor sanitation in a hospitable climate creates a ripe environment for spread of many NTDs. These conditions are generally exacerbated since displaced populations frequently lack access to health care through national public health programs (e.g., immunization programs, PCT, established community-based interventions).

## Why Are Displaced Populations Frequently Excluded from Integrated NTD Control Programs?

The current integrated NTD control programs are supported by multiple partners (e.g., USAID, pharmaceutical industry, WHO) and have been tremendously successful, reaching more than 1 billion people worldwide. However, these programs are successful in some locations and falter in others, and this relates to local dynamics and issues with population movement, changing food supply, and relationships between drug distributors and targeted groups [8]. Displaced populations are frequently excluded from host government integrated control programs. The majority of programs are coordinated and run through the Ministries of Health (MOH) at the country level. Ministries and public policy makers are faced with limited funds and will generally focus on established priorities and spend resources on their own native population/constituents. This occurs due to structural barriers. For example, there is little incentive for country ministries to spend their limited resources on displaced populations since they are considered temporary, are often viewed as “irregular,” and often lack legal status and political voice.

## Displaced Populations, NTDs, and the International Health Agenda

Evaluating disease burden in displaced populations and understanding the barriers to program intervention are crucial in guiding evidence based policy decisions to better reach these populations in the future. Despite the many political, logistical, and ethical barriers to conducting NTD programs, surveillance, and research in conflict regions, or in countries where “unwelcome” refugees reside, there exists an urgent need. Although limited, the existing data suggest very high burdens of NTDs in displaced populations [9,10]. For those working in the NTD community, the largely inadvertent exclusion of displaced populations needs to be of paramount concern since mobile populations present a threat to successful control and elimination of NTDs in resident populations. As a documented example, malaria elimination programs have been seen to struggle with success because of failure to address population mobility and migration [11] and it goes without saying that this same struggle exists in treating NTDs.

It is important to recognize that although there are few programs that systemically reach displaced populations, there are organizations and agencies, such as the International Organization for Migration (IOM), who are interested, capable, and willing to distribute and monitor NTD programs. Unfortunately, because of the current structure of NTD programs, many organizations have difficulty obtaining drugs or the managerial support necessary for implementing effective Mass Drug Administration (MDA) programing [12]. There are many small-scale programs carried out by organizations serving refugee populations (e.g., small non-governmental organization (NGO) programs) but these are frequently not sustainable, and tend to be focal, intermittent and often not coordinated with the larger NTD control initiatives. The United States program for refugee resettlement operates a relatively large-scale program addressing refugee populations resettling to the US. However, this is a unique program since it is specifically aimed at refugees in the US resettlement pipeline and is moving refugees from endemic to non-endemic settings. Although this program reaches more than 50,000 refugees annually, this accounts for less than 1% of the worlds displaced population.

Partnering between the NTD community and organizations serving these displaced populations would be mutually beneficial by creating opportunities to expand existing programing, and would further serve the goal of NTD control and elimination in resident communities. Collaboration would benefit the larger NTD community by allowing access to this unique population, providing the opportunity to answer questions that are difficult to answer in endemic environments. For example, treating refugees who are moving from endemic to non-endemic areas allows measurement of effectiveness of treatment (drugs and regimens) for specific NTD organisms—efficacy data that is still largely unknown. Furthermore, collaboration would create an opportunity to develop tools for the measurement of the impact and control of specific pathogens. The movement to non-endemic areas creates an opportunity to identify biologic markers of active infection that could subsequently be used to monitor the success of control in endemic areas. In addition, novel programing providing US-bound refugees with pre-migration PCT to treat strongyloidiasis represents an opportunity for the larger MDA community to evaluate the benefits of presumptive treatment for this particular potentially fatal pathogen. Furthermore, collaboration would be an act of compassion by providing displaced populations with greater access to treatment and control programs.

## Treatment and Control Programs for Refugees Are the Right Thing to Do and There Are Steps to Take

It must not be forgotten that the right to health is a basic human right. The human rights conventions provides a policy framework from which to advocate for more research in this area, legal tools for member state accountability, and platforms for action on global initiatives, such as the Millennium Development Goals. Specifically, the right to health as defined by Article 12 of the 1976 International Covenant on Economic, Social and Cultural Rights, part C is applicable to NTDs. As of January 2014, 168 of the 192 United Nations member states were party to the Covenant [13]. Finally, the Convention Relating to the Status of Refugees establishes a principle of equity whereby hosting countries should provide refugees with a similar standard of medical care to that which is routinely available to host nationals [14,15].

WHO and other international organizations have a moral obligation to use the human rights platform to advocate for expanded treatment programs and for the provision of essential services to displaced populations. One current barrier is that the donor pharmaceutical companies have restricted distribution of medications to MOH. Therefore, non-MOH entities, such as NGOs like IOM, operating outside the current system, are generally forced into purchasing medications at market rates. WHO could advocate for a small proportion of donor medication to be allowed to be distributed through other partners (e.g., UNHCR, NGOs such as the International Rescue Committee or Medecins Sans Frontieres). The global health agenda should also encourage collaboration between the more experienced partners (e.g., WHO/USAID) and these other non-MOH organizations to implement sustainable integrated control programs [5,16].

## MDGs and the Elimination of Certain NTDs Will Not Occur Unless These Populations Are Reached; Human Displacement Is an Achilles Heel

NTD treatment for displaced populations should be a priority for the global health agenda. Inclusion of these populations would decrease health disparities, increase the human right to health, and ultimately benefit the ongoing control and eradication efforts of these insidious diseases, as called for in MDG 6. As long as conflict continues and displaced people are excluded from government-led initiatives, NTD programs will struggle due to remaining pockets of ongoing transmission, introduction, and re-introduction of infection. Elimination of this disparity in disease burden is contingent upon addressing inequities in access to available medications and systematic programing. Although we have the tools, increased awareness of the exclusion of these populations and collaborative partnerships and political will is needed.
